# Quorum Sensing Down-Regulation Counteracts the Negative Impact of *Pseudomonas aeruginosa* on CFTR Channel Expression, Function and Rescue in Human Airway Epithelial Cells

**DOI:** 10.3389/fcimb.2017.00470

**Published:** 2017-11-10

**Authors:** Émilie Maillé, Manon Ruffin, Damien Adam, Hatem Messaoud, Shantelle L. Lafayette, Geoffrey McKay, Dao Nguyen, Emmanuelle Brochiero

**Affiliations:** ^1^Centre de Recherche du Centre Hospitalier de l'Université de Montréal, Montréal, QC, Canada; ^2^Département de Médecine, Université de Montréal, Montréal, QC, Canada; ^3^Meakins-Christie Laboratories at the Research Institute of the McGill University Health Centre, Montréal, QC, Canada; ^4^Department of Medicine, McGill University, Montréal, QC, Canada

**Keywords:** cystic fibrosis, *P. aeruginosa*, infection, CFTR, Vx-809, correctors, LasR, furanone

## Abstract

The function of cystic fibrosis transmembrane conductance regulator (CFTR) channels is crucial in human airways. However unfortunately, chronic *Pseudomonas aeruginosa* infection has been shown to impair CFTR proteins in non-CF airway epithelial cells (AEC) and to alter the efficiency of new treatments with CFTR modulators designed to correct the basic CFTR default in AEC from cystic fibrosis (CF) patients carrying the F508del mutation. Our aim was first to compare the effect of laboratory strains, clinical isolates, engineered and natural mutants to determine the role of the LasR quorum sensing system in CFTR impairment, and second, to test the efficiency of a quorum sensing inhibitor to counteract the deleterious impact of *P. aeruginosa* both on wt-CFTR and on the rescue of F508del-CFTR by correctors. We first report that exoproducts from either the laboratory PAO1 strain or a clinical ≪Early≫ isolate (from an early stage of infection) altered CFTR expression, localization and function in AEC expressing wt-CFTR. Genetic inactivation of the quorum-sensing LasR in PAO1 (PAO1Δ*lasR*) or in a natural clinical mutant (≪Late≫ CF-adapted clinical isolate) abolished wt-CFTR impairment. PAO1 exoproducts also dampened F508del-CFTR rescue by VRT-325 or Vx-809 correctors in CF cells, whereas PAO1Δ*lasR* had no impact. Importantly, treatment of *P. aeruginosa* cultures with a quorum sensing inhibitor (HDMF) prevented the negative effect of *P. aeruginosa* exoproducts on wt-CFTR and preserved CFTR rescue by correctors in CF AEC. These findings indicate that LasR-interfering strategies could be of benefits to counteract the deleterious effect of *P. aeruginosa* in infected patients.

## Introduction

Anion secretion through cystic fibrosis transmembrane conductance regulator (CFTR) channels expressed at the apical membrane of airway epithelial cells (AEC) is essential to maintain an adequate airway periciliary liquid volume necessary for effective mucociliary clearance, a key mechanism to clear inhaled pathogens from the lungs. Bacterial colonization and infections are however frequent in patients with chronic obstructive pulmonary disease (COPD) for example (Matkovic and Miravitlles, 2013). In cystic fibrosis (CF) airways, mutations in the *Cftr* gene lead to dysfunctional Cl^−^ and ${\text{HCO}}_{3}^{-}$ secretion associated with reduced periciliary liquid volume, impaired mucociliary clearance, accumulation of viscous mucus and airway surface liquid acidification. These phenomena, in turns, favor bacterial infections (Haq et al., 2015). The prevalence of respiratory pathogens varies among patients and over the course of the disease. However, ultimately most CF patients are chronically colonized by *Pseudomonas aeruginosa* (*P. aeruginosa*). This pathogen readily adapts to its environment and displays extensive genotypic and phenotypic changes over the course of chronic infection in the human lung (Folkesson et al., 2012). *P. aeruginosa* isolates from CF patients frequently exhibit host-adapted mutations, including in the *lasR* gene. LasR is the primary transcriptional regulator for quorum sensing, a bacterial communication system that allows for coordinated gene expression, including genes involved in virulence factor production (Feliziani et al., 2010; Jimenez et al., 2012; Qin et al., 2012; Rutherford and Bassler, 2012).

Exposure to *P. aeruginosa* bacteria and/or *P. aeruginosa* secreted exoproducts has been associated with loss of epithelial integrity *in vitro* and with progressive tissue damage and lung function decline *in vivo* (Coraux et al., 2004; Rejman et al., 2007; Folkesson et al., 2012). In addition, we recently showed that exoproducts under LasR control impair airway epithelial repair after injury (Ruffin et al., 2016). Interestingly, quorum sensing inhibitors have been identified within libraries of natural and chemical compounds and have been proposed as adjuvants to antibiotics based on their capability to restrain biofilm formation and bacterial virulence, without altering bacterial growth (Bhardwaj et al., 2013; Kalia, 2013). Our recent work provided the first proof of concept that a quorum sensing inhibitor, namely the 4-hydroxy-2, 5-dimethyl-3(2H)-furanone (HDMF; Choi et al., 2014; also known as furaneol or strawberry furanone) abrogates the deleterious effect of *P. aeruginosa* on the repair of CF and non-CF airway epithelia (Ruffin et al., 2016).

*P. aeruginosa* infections not only play a detrimental role on airway integrity but also impacts CFTR expression and function. Indeed, several studies, including from our group, showed that exposure to *P. aeruginosa* strains or exoproducts reduced the expression of wt-CFTR at the apical membrane and CFTR-mediated Cl^−^ secretion through non-CF human AEC (Swiatecka-Urban et al., 2006; Rubino et al., 2014; Trinh et al., 2015). Such reduction of CFTR function in chronically infected airways may impair mucociliary clearance and consequently microbial clearance, even in non-CF airways, such as in COPD patients. The mechanisms by which *P. aeruginosa* alters CFTR is likely complex. Our data indicated that exposure to *P. aeruginosa* exoproducts reduced CFTR protein synthesis and enhanced CFTR protein degradation in AEC (Trinh et al., 2015). It has been recently reported that LasB elastase down-regulates wt-CFTR protein levels (Saint-Criq et al., 2017). Enhanced wt-CFTR ubiquitination and degradation, at least in part due to a CFTR inhibitory factor (Cif, present in *P. aeruginosa* outer membrane vesicles) has also been reported (Bomberger et al., 2011). The inhibition of CFTR endocytic recycling could also be mediated by a NHERF-dependent mechanism (Rubino et al., 2014).

During the last decade, the development of new mutation-specific CF therapeutics, with small molecules have given hope to rescue the basic CFTR defect (for review Bell et al., 2015). Specifically, a first CFTR potentiator (Vx-770, Kalydeco^MD^) has been approved for patients with several class III (characterized by improper channel gating/regulation) and class IV (decreased channel conductance) mutations. In addition, several molecules called correctors (including VRT-325 and Vx-809) (Pedemonte et al., 2005; Goor et al., 2006; Van Goor et al., 2011) are directed against class II mutations, including the most frequent, F508del (characterized by improper CFTR protein folding, trafficking, membrane stability and function). Although CFTR correctors allow partial F508del-CFTR maturation and functional rescue *in vitro*, the first clinical trials with Vx-809 did not show significant changes in lung function (Brewington et al., 2015). Interestingly, a corrector/potentiator combined treatment (Vx-809+Vx-770) elicits more benefits (Wainwright et al., 2015) and this combination (Orkambi™) has recently been approved for homozygous F508del CF patients. However, clinical studies on CFTR modulators demonstrated that the response to treatments is variable among patients and the beneficial effects on lung function remained less than expected (Brewington et al., 2015). The cause of the limited and variable efficiency of CFTR modulators remains unclear and may be multiple, including modifier genes (Strug et al., 2016), disease severity, environmental factors, etc. In addition, work from our laboratory and others unveiled that *P. aeruginosa* infection restrains the functional rescue of F508del-CFTR by VRT-325 and Vx-809 correctors, alone or in combination with the Vx-770 potentiator (Stanton et al., 2015; Trinh et al., 2015). However, the bacterial products responsible for the deleterious effect of *P. aeruginosa* on CFTR rescue remain to be defined. Moreover, a strategy using quorum sensing inhibitors to decrease the production of virulence factors has, to the best of our knowledge, never been carry out in an attempt to counteract the harmful effect of *P. aeruginosa* on CFTR rescue impairment.

The aim of our study was first to define if *P. aeruginosa* exoproducts from clonally-related early and late-adapted clinical isolates elicit the same effect on wt-CFTR expression, localization and function. Then, using a wt-laboratory strain and an engineered *lasR* mutant, we tested the hypothesis that *P. aeruginosa* LasR quorum sensing play a role in wt-CFTR and F508del-CFTR rescue impairment. Finally, experiments were undertaken to evaluate the efficiency of a quorum sensing inhibitor to counteract the deleterious effect of *P. aeruginosa* on wt-CFTR and on the rescue of F508del-CFTR.

## Materials and methods

### Culture of CFBE-ΔF508 and CFBE-wt cell lines

CFBE-ΔF508 and CFBE-wt cell lines [CFBE41o- parental cells (Kunzelmann et al., 1993) stably transduced, respectively, with F508del-*Cftr* and wt-*Cftr* Bebok et al., 2005] were grown in EMEM medium (Wisent Inc., St-Bruno, QC, CA) supplemented with 10% FBS (Life technologies, Burlington, QC, CA), 2 mM L-glutamine (Life technologies) and 100U/ml of penicillin-streptomycin (Life technologies) on 35 mm petri dishes (Corning Inc., Corning, NY, USA), Lab-Tek 8 chamber slides (Thermo-Fisher Scientific Inc., Waltham, MA, USA) and permeant filters (4.67 cm^2^; Corning Inc.) coated with a LHC basal medium (Life technologies) solution containing 1 mg/ml BSA (Life technologies), 0.05 mg/ml bovine collagen I (Life technologies) and 1 mg/ml human fibronectin (VWR, Mont-Royal, QC, CA). Cells were cultured for 5 days on Lab-Tek chamber slides for immunofluorescence assays and 8 days on petri dishes before protein extraction, while cells on permeant filters were cultured for 3 weeks before electrophysiological measurements (Trinh et al., 2012, 2015; Bilodeau et al., 2016).

### Culture of primary human airway epithelial cells

CF primary human AEC were isolated from bronchial tissues collected from CF patients (homozygous for the F508del mutation) who underwent a lung transplantation at CHUM hospital according to approved ethical protocols and with written informed consents, in accordance with the Declaration of Helsinki. After dissection, tissues were rinsed few times with sterile PBS and then incubated overnight on a rocking platform at 4°C with MEM medium (Life technologies) supplemented with 7.5% NaHCO_3_ (Sigma-Aldrich, Saint-Louis, MO, USA), 2 mM L-glutamine, 10mM HEPES (Thermo-Fisher Scientific Inc., #SH3023701), 0.05 mg/ml gentamycin, 50U/ml penicillin-streptomycin, 0.25 μg/ml Fungizone (Life technologies) and containing 0.1% protease (from *Streptomyces griseus*; Sigma-Aldrich) and 10 μg/ml DNAse (Deoxyribonuclease I from bovine pancreas; Sigma-Aldrich). The protease-DNAse activity was then neutralized with FBS, cells gently scraped off the remaining tissue and red blood cells removed by treatment with ACK lysis solution (0.1 mM NH_4_Cl, 10 μM KHCO_3_, 10 nM EDTA). After counting, cells were seeded on flasks coated with Purecol (Cedarlane Laboratory, Burlington, ON, CA) and cultured in CnT-17 medium (CellnTec Advanced Cell systems, Bern, CH) until confluency is reached. Cells were then detached with trypsin solution, seeded on permeant filters (1.1 cm^2^; Corning) coated with collagen IV (Sigma-Aldrich) and cultured in CnT-17 until confluency (~5 days). The apical medium was then removed to create an air-liquid interface and the basolateral medium was replaced by a differentiation medium (1:1 volume of BEGM (Lonza, Basel, CH) and DMEM (Life technologies) supplemented with 1.5 μg/ml BSA, 1 × 10^−7^ M retinoic acid and 100U/ml of penicillin-streptomycin) every 2 days for at least 35 days to obtain highly differentiated cultures (Trinh et al., 2012, 2015; Bilodeau et al., 2016; Ruffin et al., 2016).

### *P. aeruginosa* strains, growth condition and preparation of diffusible material

Genotypic and phenotypic characteristics of *P. aeruginosa* strains used in this study are presented in Table 1. Two clonally related clinical isolates from the same CF patient recovered at 6 months of age (Early) and 8 years (Late) were used (Burns et al., 2001; Smith, 2006). Their whole genome have been sequenced and 68 mutations were identified in the Late isolate, including a nonsense mutation in the *lasR* gene (Burns et al., 2001; Smith, 2006). In addition, the common laboratory wild-type (wt) *P. aeruginosa* strains PAO1 (Nguyen et al., 2011) and PAO1-V [characterized by increased protease production compared with PAO1, Hobden, 2002], and the isogenic PAO1-V Δ*lasR* mutant (Hobden, 2002) were included in our study.

**Table 1:** Genotypic and phenotypic characteristics of *P. aeruginosa* strains.

**Strain**	**Relevant genotypic and phenotypic characteristics**	**References**
Early (E)	AMT0023-30 *P. aeruginosa* clinical isolate from a 6-month-old CF patient, with wild-type *lasR* allele	Burns et al., 2001; Smith, 2006
Late (L)	AMT0023-34 *P. aeruginosa* clinical isolate clonally related to the Early strain, recovered from the same patient at age 8. 1-bp deletion in *lasR* at nucleotide 147 leading to a non-sense mutation	Burns et al., 2001; Smith, 2006
PAO1	*P. aeruginosa* wild-type laboratory strain	Nguyen et al., 2011
PAO1-V	*P. aeruginosa* wild-type strain, variant of PAO1 with high basal expression of proteases	Hobden, 2002
PAO1-VΔ*lasR*	*lasR*::Gm^R^ mutation in *lasR* gene in PAO1-V parental *P. aeruginosa* strain	LaFayette et al., 2015

Bacterial strains were grown on Luria Bertani (LB, Difco, Montreal, QC, CA) 1.5% agar plates overnight (when needed, gentamicin (50 μg/ml) was used for bacterial selection), and single colonies were used to inoculate LB medium. Where indicated, LB medium was supplemented with 0.125 mg/ml HDMF (Sigma-Aldrich) and viable bacteria counts were determined by standard microdilution and colony forming units plate counting (Choi et al., 2014). To obtain the *P. aeruginosa* exoproducts, planktonic bacterial cultures, in the absence or presence of HDMF, were grown for 72 h with agitation at 250 rpm at 37°C, and then centrifuged at 7,200 g for 10 min at room temperature. Supernatants were filtered with low-protein binding 0.22 μm cellulose acetate filters (Corning) and aliquots of exoproducts were stored at −80°C, as previously done (Ruffin et al., 2016).

### Immunoblotting

CFBE cells were scraped in ice-cold PBS, cell suspensions were centrifuged at 12,000 rpm for 5 min at 4°C and cell pellets were then solubilized in RIPA lysis buffer [150 mM NaCl, 20 mM Tris-HCl pH 8.0, 1% Triton X-100, 0.08% deoxycholic acid, 0.1% SDS, and protease inhibitor cocktail (≪complete mini EDTA free≫ Roche Diagnostic, Laval, QC, CA)] for 20 min on ice. After centrifugation (12,000 rpm, 15 min at 4°C), the supernatants were collected and protein concentration was quantified by Bradford assay. Cell lysates in 2X sample buffer [62.5 mM Tris-HCl pH 6.8, 2% SDS, 0.2% bromophenol blue, 10% glycerol and 40% β-mercapto-ethanol] were separated by SDS-PAGE (7.5%) and transferred onto nitrocellulose membranes. The membranes were first blocked with 10% dried fat-free milk in TBS-Tween (TBS-T) for 1 h at room temperature, then the upper part of the membrane was incubated for 18 h at 4°C with the polyclonal anti-CFTR 596 antibody (dilution 1:1,000, Cystic Fibrosis Foundation Therapeutics (CFFT), Bethesda, MA, USA) in TBS-T supplemented with 5% bovine serum albumin (BSA). The lower part of the membrane was incubated with purified rabbit anti-Pan-actin polyclonal antibody [Cell Signaling Danvers, MA, USA, dilution 1:1,000 in TBS-T supplemented with 5% bovine serum albumin (BSA, Sigma Aldrich)], to ensure equivalent loading and for signal normalization. Membranes were washed 3 × 15 min with TBS-T and then incubated for 1 h with goat anti-mouse HRP conjugated antibody (upper membrane; dilution 1:1,000; Millipore, Etobicoke, ON, CA) and goat anti-rabbit HRP conjugated antibody (lower membrane; dilution 1:1,000, Cell Signalling). Finally, the membranes were washed 3 × 15 min with TBS-T before chemiluminescent (ECL) detection (GE Healthcare Life Sciences, Baie d'Urfe, QC, CA). The anti-CFTR antibody recognized a 180 kDa (mature band C) and a 140–160 kDa (immature band B) protein and the intensity of bands was quantified with Multi Gauge software (Trinh et al., 2015). Each quantified CFTR band density was normalized (divided) by the value of actin density, for the same condition. Finally, the ratio (CFTR/actin) in each condition was presented as % of the control (LB) condition.

### Immunofluorescence

CFBE-wt cells seeded on Lab-Tek 8 chamber slides (Thermo-Fisher Scientific) were fixed in 4% paraformaldehyde for 20 min and permeabilized with 0.1% triton X100 for 10 min at room temperature (Trinh et al., 2015). After blocking in PBS containing 10% fetal bovine serum (FBS) for 1 h, cells were first incubated with the polyclonal anti-CFTR 596 antibody (dilution 1:250, CFFT) overnight at 4°C, then with Alexa Fluor 488 conjugated anti-mouse antibody for 1 h at RT (dilution 1:200, Life Technologies Inc.). Slides were finally rinsed and counterstained with DAPI (dilution 1:1,000, Life Technologies). Fluorescence images were captured by an epifluorescent Olympus microscope. Absence of background signal and non-specific staining was verified in control experiments by omitting primary or secondary antibodies.

### Electrophysiology

Primary CF human AEC and immortalized CFBE cells were cultured on permeable filters at the air-liquid interface. Where indicated, CFBE-ΔF508 and primary CF human AEC were treated (at the basolateral side) for 24 h before electrophysiological measurements with a CFTR corrector (VRT-325 (CFFT) or Vx-809 (Selleckchem, Houston, TX, USA), 5 μM). One hour prior to short-circuit current *I*_*sc*_ measurements in an Ussing chamber, the apical side was submerged with their specific cell culture medium (see CFBE and primary cell culture sections). After washing, the filters were then mounted in a heated (37°C) Ussing chamber. To evaluate CFTR Cl^−^ current through apical membranes, a Cl^−^ gradient was established (low Cl^−^ physiological solution at the apical side and high Cl^−^ solution at the basolateral side) and the basolateral membranes were permeabilized with amphotericin B (7.5 μM). Transepithelial potential difference was clamped to zero by an external voltage clamp amplifier (VCCMC2, Physiological Instruments, San Diego, CA, USA) with KCl agar-calomel half-cells and Ag-AgCl electrodes, and the resulting *I*_*sc*_ was recorded continuously on a computer with PowerLab system (ADInstruments, Toronto, ON, CA). Membrane resistance was verified with 1 mV pulses every 10 s (Trinh et al., 2015; Bilodeau et al., 2016).

### Statistical analysis

**Figure 1:**
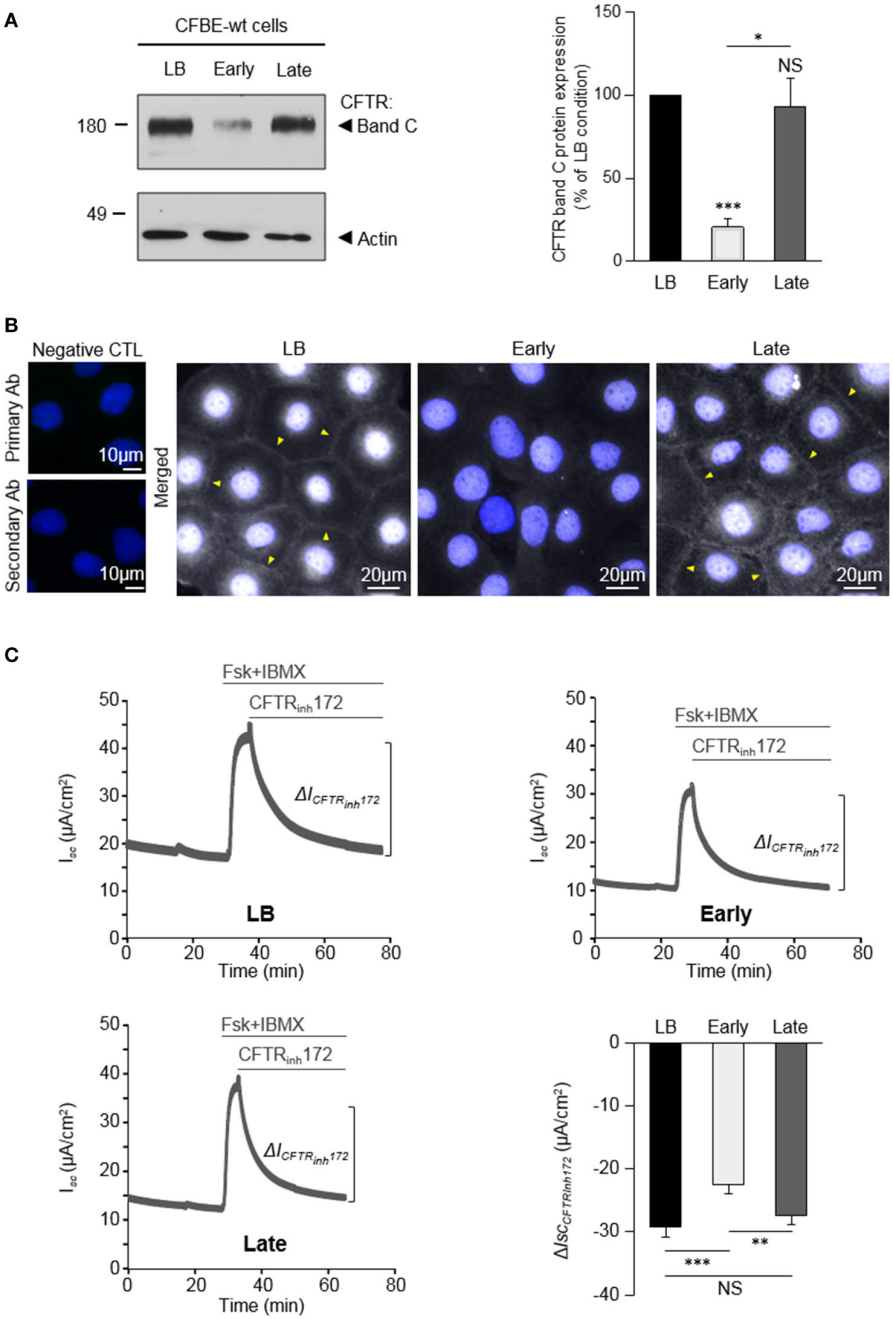
Variable impact of *P. aeruginosa* exoproducts from Early and Late clinical isolates on CFTR expression, localization and function. CFBE-wt cells were challenged for 24 h with exoproducts from the Early or Late isolates, or LB medium (control condition). **(A)** Representative immunoblots (left) and densitometric analysis (right, reported as % of LB control) of mature (band C) CFTR protein expression levels (*n* = 6). **(B)** Representative immunofluorescence images (of *n* = 3 independent experiments) of CFBE-wt cells treated with LB, Early or Late. CFTR was detected with an anti-CFTR antibody (Ab) coupled to Alexa Fluor 488 conjugated anti-mouse antibody. Nuclei were stained with DAPI (blue) (*n* = 3). **(C)** Representative traces of short-circuit current (*I*_*sc*_) measurements in Ussing chamber and quantification of mean CFTR_Inh172_ (20 μM)-sensitive currents (Δ*I*_CFTRInh−172_) through CFBE-wt cells, in each condition. (*n* = 12). ^*^p < 0.05, ^**^p < 0.01, ^***^p < 0.001.

**Figure 2:**
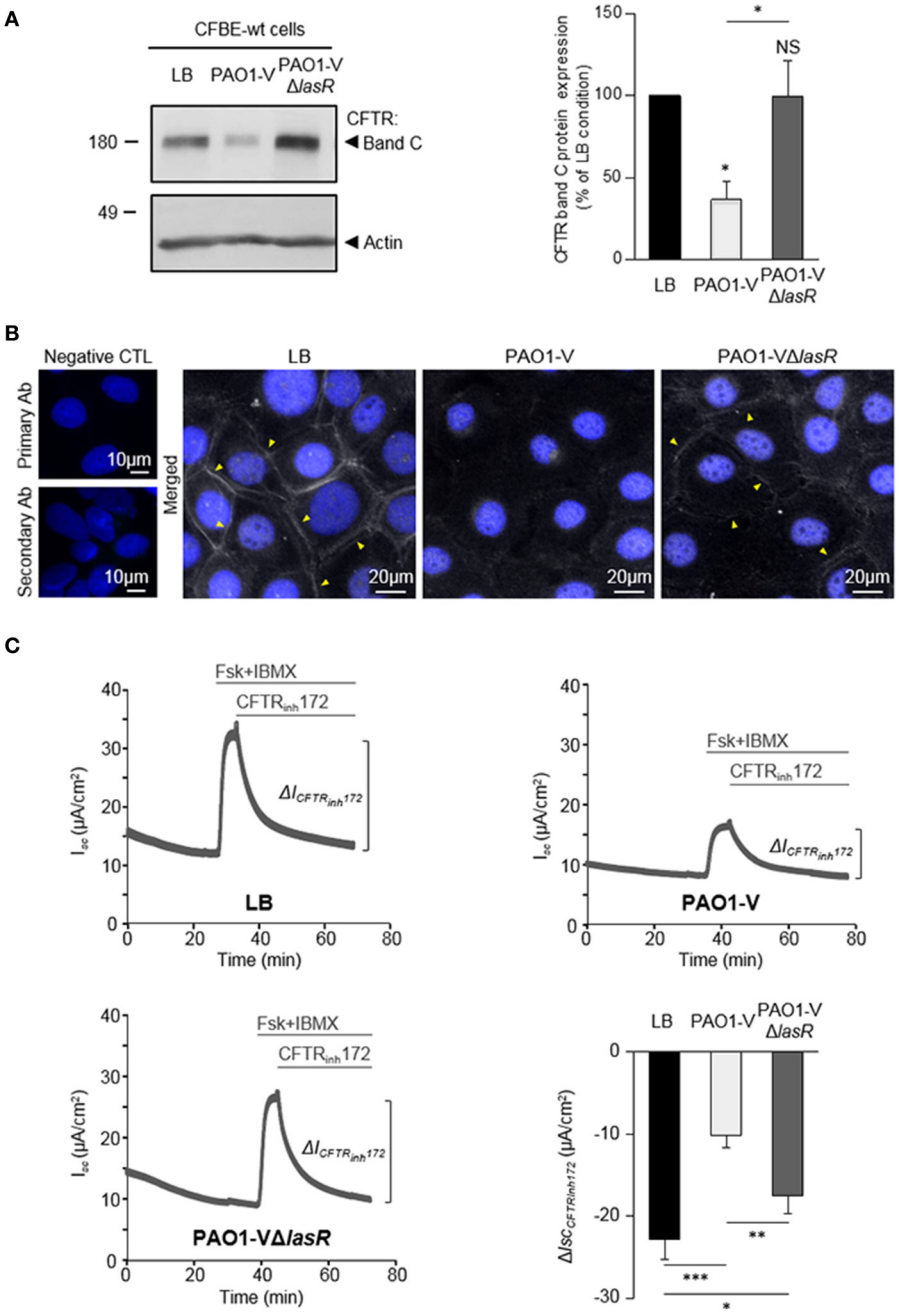
Role of LasR quorum sensing in the deleterious effect of *P. aeruginosa* on CFTR expression, localization and function. CFBE-wt cells were treated for 24 h with LB or exoproducts from PAO1-V or PAO1-V mutated in the *lasR* gene (PAO1-VΔ*lasR*). **(A)** Representative immunoblots (left) and densitometric analysis (right, reported as % of LB control) of mature (band C) CFTR protein expression levels (*n* = 6). **(B)** Representative immunofluorescence images (of *n* = 4 independent experiments) of CFBE-wt cells treated with LB, PAO1-V or PAO1-VΔ*lasR* exoproducts. CFTR was detected with an anti-CFTR antibody (Ab) coupled to Alexa Fluor 488 conjugated anti-mouse antibody. Nuclei were stained with DAPI (blue). **(C)** Representative traces of short-circuit current (*I*_*sc*_) measurements in Ussing chamber and quantification of mean CFTR_Inh172_ (20 μM)-sensitive currents (Δ*I*_CFTRInh−172_) through CFBE-wt cells, in each condition. (*n* = 9). NS, non-significant, ^*^p < 0.05, ^**^p < 0.01, ^***^p < 0.001.

**Figure 3:**
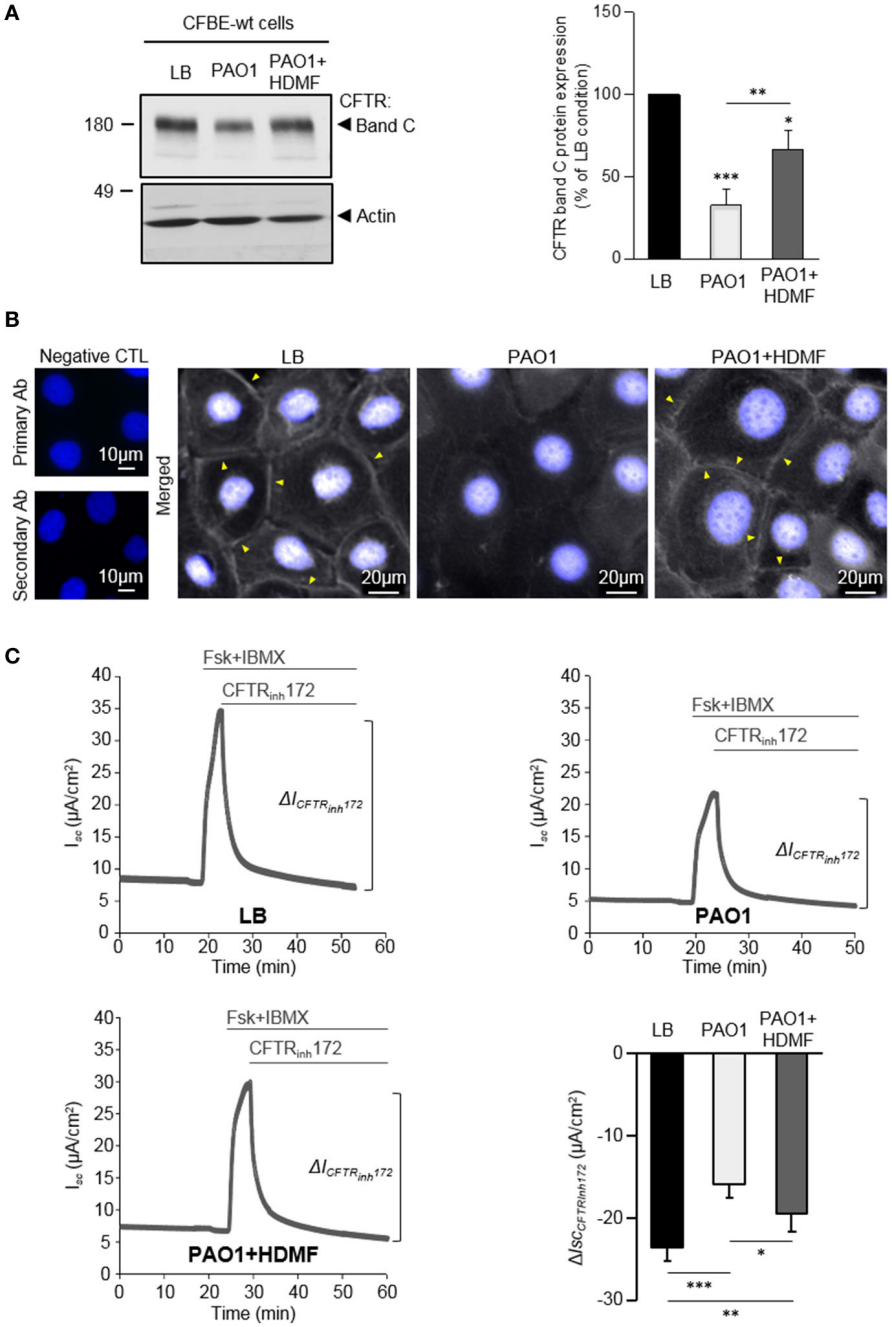
Quorum sensing inhibition with HDMF counteracts the negative effect of *P. aeruginosa* on CFTR expression, localization and function. PAO1 bacteria were grown in LB with or without 0.125 mg/ml HDMF prior to collection of the bacterial filtrates. CFBE-wt cells were then treated for 24 h with exoproducts from PAO1 cultured with (PAO1+HDMF) and without HDMF (PAO1). LB medium was used as control. **(A)** Representative immunoblots (left) and densitometric analysis (right, reported as % of LB control) of mature (band C) CFTR protein expression levels (*n* = 6). **(B)** Representative immunofluorescence images (of *n* = 4 independent experiments) of CFBE-wt cells treated with LB, PAO1, or PAO1+HDMF exoproducts. CFTR was detected with an anti-CFTR antibody (Ab) coupled to Alexa Fluor 488 conjugated anti-mouse antibody. Nuclei were stained with DAPI (blue). **(C**) Representative traces of short-circuit current (*I*_*sc*_) measurements in Ussing chamber and quantification of mean CFTR_Inh172_ (20 μM)-sensitive currents (Δ*I*_CFTRInh−172_) through CFBE-wt cells, in each condition (*n* = 10). NS, non-significant, ^*^p < 0.05, ^**^p < 0.01, ^***^p < 0.001.

**Figure 4:**
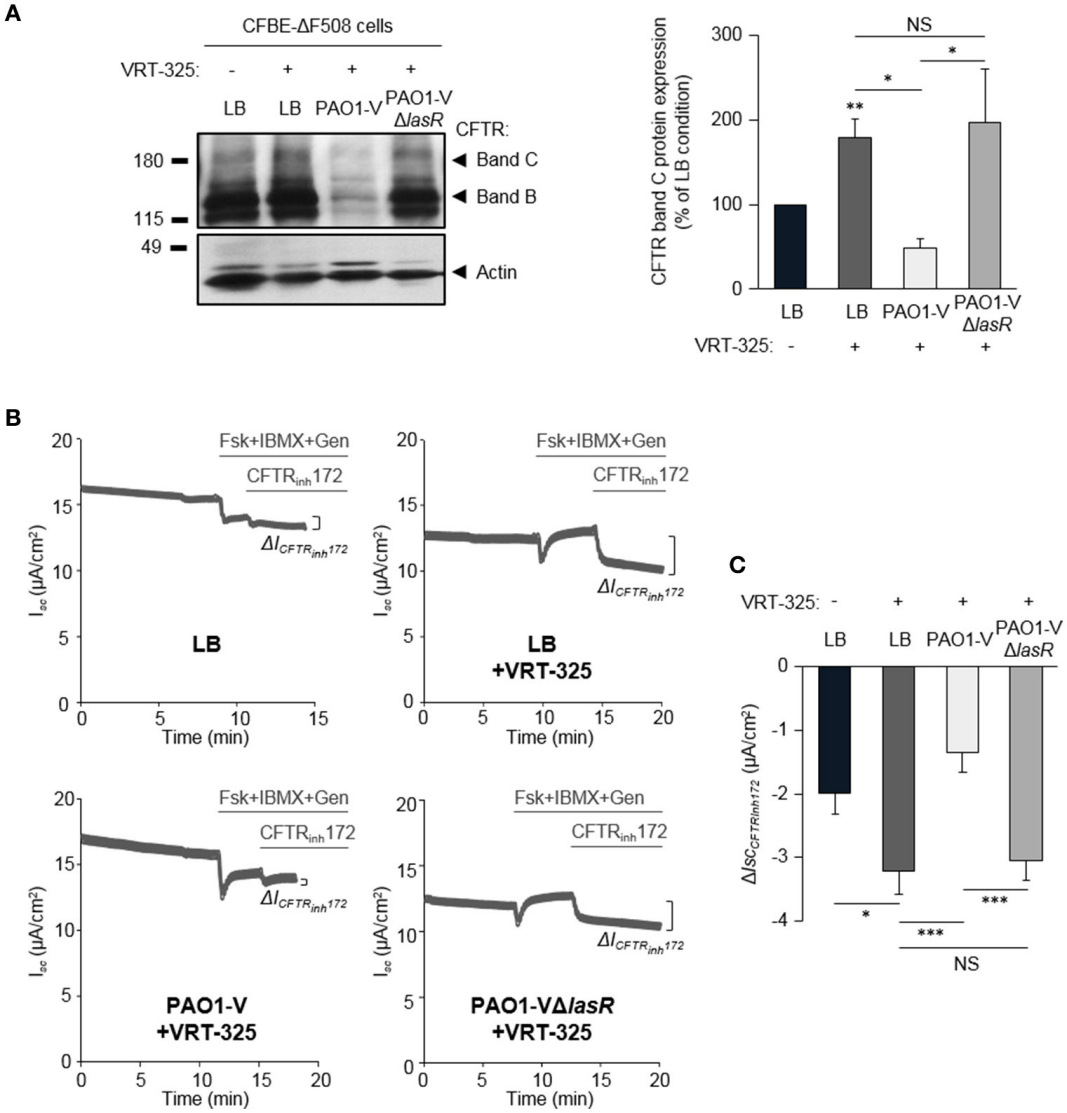
Role of LasR quorum sensing in the deleterious effect of *P. aeruginosa* on F508del-CFTR maturation and functional rescue by VRT-325. CFBE-ΔF508 cells treated or not with the VRT-325 corrector (5 μM) were exposed to LB or exoproducts from PAO1-V or PAO1-V mutated in the *lasR* gene (PAO1-VΔ*lasR*) for 24 h. **(A)** Representative immunoblots (left) and densitometric analysis (right, reported as % of LB control) of mature (band C) CFTR protein expression levels (*n* = 10). **(B**) Representative traces of short-circuit current (*I*_*sc*_) measurements in Ussing chamber and quantification of mean CFTR_Inh172_ (20 μM)-sensitive currents (Δ*I*_CFTRInh−172_) **(C)** through CFBE-ΔF508 cells, in each condition (*n* = 13). NS, non-significant, ^*^p < 0.05, ^**^p < 0.01, ^***^p < 0.001.

**Figure 5:**
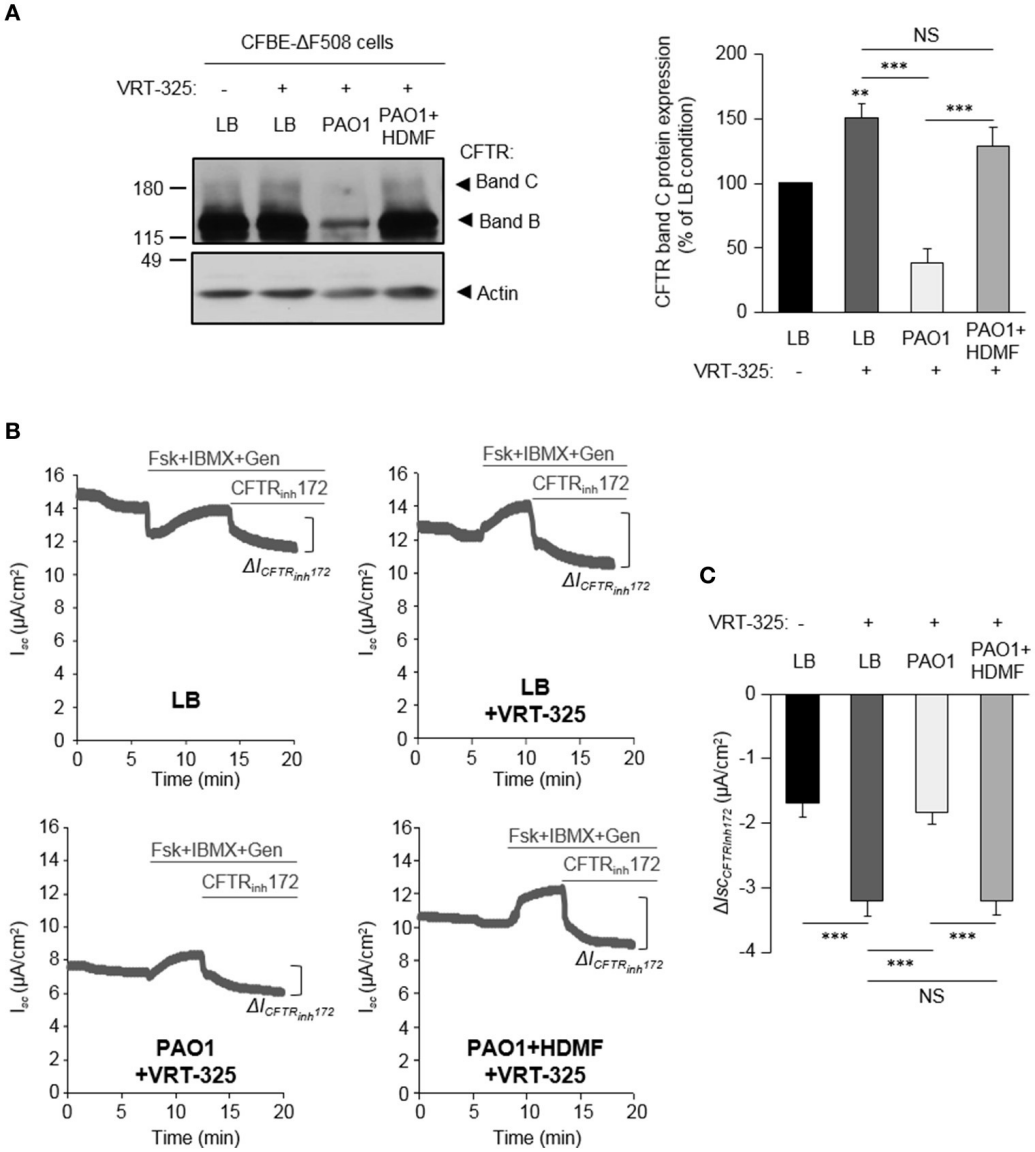
Quorum sensing inhibition abrogates the negative effect of *P. aeruginosa* on F508del-CFTR maturation and functional rescue with VRT-325. CFBE-ΔF508 cells were treated or not, for 24 h, with VRT-325 and LB or exoproducts from PAO1 bacteria cultured, or not, with 0.125 mg/ml of HDMF. **(A)** Representative immunoblots (left) and densitometric analysis (right, reported as % of LB control) of mature (band C) CFTR protein expression levels (*n* = 7). **(B**) Representative traces of short-circuit current (*I*_*sc*_) measurements in Ussing chamber and quantification of the mean CFTR_Inh172_ (20 μM)-sensitive currents (Δ*I*_CFTRInh−172_) **(C)** through CFBE-ΔF508 cells, in each condition (*n* = 9). NS, non-significant, ^**^p < 0.01, ^***^p < 0.001.

**Figure 6:**
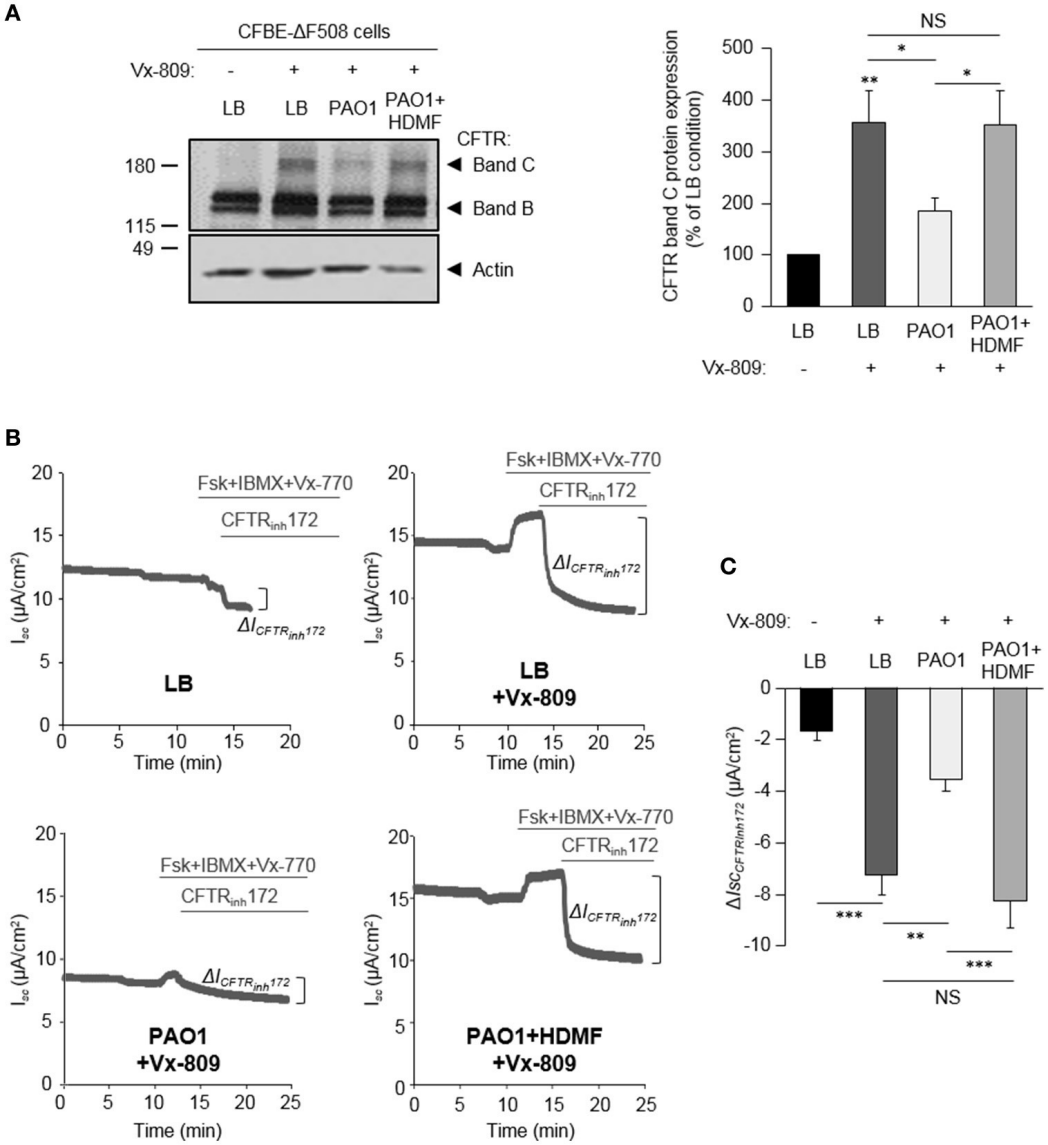
Quorum sensing inhibition abrogates the effect of *P. aeruginosa* on F508del-CFTR maturation and functional rescue by Vx-809. CFBE-ΔF508 cells were treated or not, for 24 h, with the Vx-809 corrector (5 μM) and LB or exoproducts from PAO1 bacteria cultured, or not, in the presence of 0.125 mg/ml of HDMF. **(A)** Representative immunoblots (left) and densitometric analysis (right, reported as % of LB control) of mature (band C) CFTR protein expression levels (*n* = 11). **(B)** Representative traces of short-circuit current (*I*_*sc*_) measurements in Ussing chamber and quantification of mean CFTR_Inh172_ (20 μM)-sensitive currents (Δ*I*_CFTRInh−172_) **(C)** through CFBE-ΔF508 cells, in each condition (*n* = 5). NS, non-significant, ^*^p < 0.05, ^**^p < 0.01, ^***^p < 0.001.

**Figure 7:**
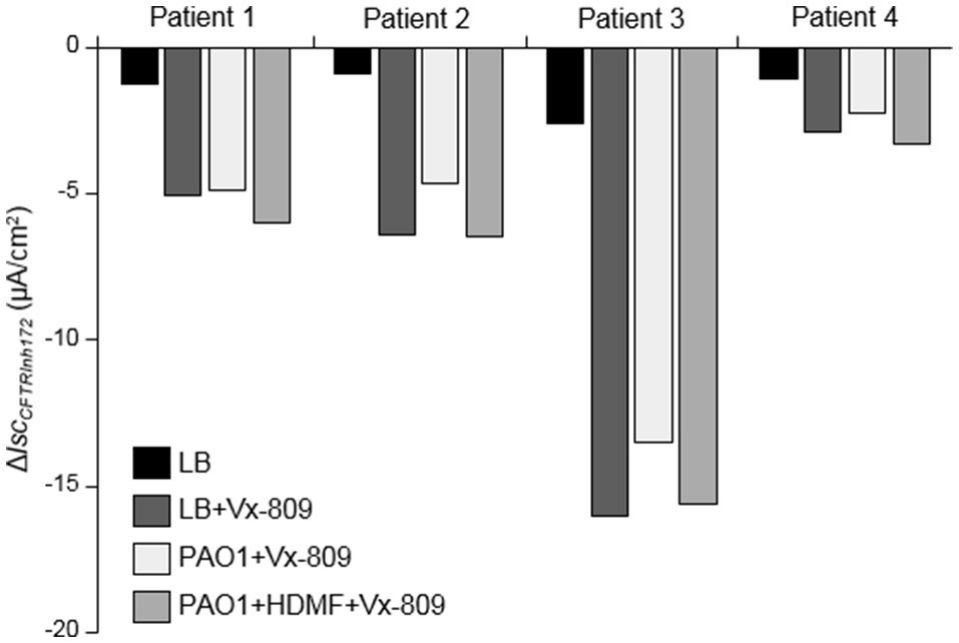
Improved CFTR rescue by Vx-809 in primary airway epithelial cells from CF patients with F508del mutations after *P. aeruginosa* quorum sensing inhibition. Short-circuit currents measurements were performed on human primary airway cells from 4 different CF patients homozygous for the F508del mutation cultured at the air-liquid interface for 35 days and then treated, or not, for 24 h with 5 μM Vx-809 and LB or exoproducts from PAO1 cultured with (PAO1+HDMF+Vx-809) and without (PAO1+Vx-809) 0.125 mg/ml HDMF. LB was used as control (LB and LB+Vx-809 conditions). CFTR_Inh172_ (20 μM)-sensitive currents (Δ*I*_CFTRInh−172_), in each condition, are presented individually, for each patient.

Data are presented as mean ± SEM (Figures 1–**6**), except CFTR current measurements in primary airway epithelial cell cultures (**Figure 7**), which are presented individually for the 4 different patients tested. The number of repeated experiments is indicated in the figure legends. GraphPad Prism version 5.03 for Windows (GraphPad Software, San Diego, CA, USA, www.graphpad.com) was used to analyze all results. Paired *t*-tests were used to compare two groups as appropriate. One-way ANOVA were used for comparison of more than two groups and followed by Bonferroni's multiple *post-hoc* tests. *P*-values lower than 0.05 were considered to be significant. Non-significant (NS) and statistically significant differences with *p* < 0.05 (^*^), *p* < 0.01 (^**^), and *p* < 0.001 (^***^) are indicated in the figures.

## Results

### The impact of *P. aeruginosa* on wt-CFTR varies as a function of bacterial strains

We recently demonstrated that exoproducts from *P. aeruginosa* modulate wt-CFTR expression and function in AEC (Trinh et al., 2015). However, the consequences of *P. aeruginosa* infection on CFTR may vary as a function of *P. aeruginosa* strains. Indeed, *P. aeruginosa* strains undergo adaptive genotypic and phenotypic changes during chronic infections in CF patients, including in the production of diffusible factors (Burns et al., 2001; Smith, 2006). We therefore tested the effect of the two clonally related isolates [Early and Late (Table 1) isolates Smith, 2006], which have been collected from the same CF patient at ages of 6 months (early stage of intermittent infection) and 8 years (chronic infection with CF-adapted isolates). CFBE-wt cells were stimulated for 24 h with LB medium (control condition) or *P. aeruginosa* exoproducts from the Early or Late isolates. As shown on Figure 1A, the level of mature CFTR (band C) expression was reduced by 79% after exposure to exoproducts from the Early isolate, whereas the level of band C CFTR expression measured after exposure to the Late exoproducts was similar to the one measured in LB condition.

The impact of Early and Late *P. aeruginosa* exoproducts on CFTR localization in CFBE-wt cells was then evaluated by immunofluorescence microscopy (Figure 1B). Results showed that both the intensity of intracellular CFTR staining and the presence of CFTR at the membrane (arrow heads), as observed in LB control conditions, were severely altered by exposure to exoproducts from the Early isolate but not from the Late isolate.

Finally, the effects of *P. aeruginosa* exoproducts on CFTR function were assessed in polarized CFBE-wt cells stimulated with Early or Late exoproducts, or LB controls, for 24 h before short-circuit (*I*_*sc*_) measurements in an Ussing chamber. To specifically measure apical CFTR Cl^−^ currents, the basolateral membranes were permeabilized and a basolateral-to-apical Cl^−^ gradient was applied before addition, in the apical compartment, of forskolin (Fsk) and 3-isobutyl-1-methylxanthine (IBMX), followed by the CFTR_inh172_ (see representative traces in Figure 1C, in LB, Early and Late conditions). Our data show that the CFTR_inh172_-sensitive currents (Δ*I*_*CFTRinh*172_) are significantly reduced after treatments with exoproducts from the Early isolate (−22.38 ± 1.43 μA/cm^2^), compared to LB (−29.09 ± 1.79 μA/cm^2^). Cell cultures treated with exoproducts from the Late isolate exhibited significantly higher currents (−27.43 ± 1.36 μA/cm^2^) than cells treated with the Early isolate, reaching values similar to the control condition (LB).

Altogether, these data indicated that early intermittent infections with wt *P. aeruginosa* and chronic infections with Late CF-adapted strains may not elicit the same effects on CFTR expression, localization and function.

### Role of LasR quorum sensing system in the deleterious effect of *P. aeruginosa* on wt-CFTR

To determine whether LasR quorum sensing system play a role in the deleterious effect of *P. aeruginosa* on CFTR, we compared the impact of exposure to exoproducts from a Δ*lasR* engineered mutant (PAO1-VΔ*lasR*) to its isogenic wild-type parental strain PAO1-V. Whereas PAO1-V exoproducts severely reduced CFTR expression (significantly decreased level of mature band C, Figure 2A) and altered CFTR membrane localization (lack of CFTR membrane staining in immunofluorescence experiments, Figure 2B), this negative effect was no longer observed with the PAO1-VΔ*lasR* mutant (Figures 2A,B). Similarly, CFBE-wt cells exposed to PAO1-VΔ*lasR* exoproducts exhibited significantly higher CFTR Cl^−^ currents than cells exposed to the PAO1-V strain (Figure 2C), indicating that LasR plays a key role in CFTR impairment.

Our data indicating that LasR-regulated exoproducts are key to the damaging action of *P. aeruginosa* on CFTR, prompted us to assess the efficiency of HDMF (4-hydroxy-2, 5-dimethyl-3(2H)-furanone), a quorum sensing inhibitor known to decrease *P. aeruginosa* exoproduct production and virulence (Choi et al., 2014; Ruffin et al., 2016). Accordingly, we previously showed that the elastase production, which is directly regulated by the LasR quorum sensing, by PAO1 grown in presence of HDMF (0.125 mg/ml) was strongly reduced. As expected from a quorum sensing inhibitor, we also verified that this dose of HDMF did not affect bacterial growth (Ruffin et al., 2016). We now compared the impact of exoproducts from PAO1 grown in the absence or presence of HDMF [harboring a 90% decrease in elastase activity, Supplementary Figure S1A)] on CFTR in CFBE-wt cells. We then found that quorum sensing inhibition of wild-type PAO1 with HDMF significantly mitigated the negative impact of *P. aeruginosa* on the level of mature CFTR expression (band C, Figure 3A), membrane localization (Figure 3B) and restored CFTR function (CFTR_Inh72_-sensitive currents, Figure 3C), to levels similar to those observed in the LB condition. In control experiments, we also verified that HDMF itself does not exert any effect on CFTR protein expression (Supplementary Figure S1B). These data provide the first proof of concept that quorum sensing inhibitors may be useful tools to prevent the deleterious effect of *P. aeruginosa* on CFTR.

### Quorum sensing inhibition abrogates the negative impact of *P. aeruginosa* on F508del-CFTR maturation and functional rescue by correctors

Our previous work revealed that the ability of the CFTR corrector VRT-325 to improve F508del-CFTR maturation (switch from immature band B to partial band C maturation) and partial functional rescue of CFTR_Inh172_-sensitive CFTR currents were severely dampened by *P. aeruginosa* exoproducts from a clinical isolate (PACF508) (Trinh et al., 2015). Our data now confirmed that the maturation (restoration of a mature band C, Figure 4A) of F508del-CFTR in VRT-325 treated CFBE-ΔF508 cells (LB+VRT-325) are disrupted in the presence of PAO1-V exoproducts (PAO1-V+VRT-325). This lack of mature protein addressed to the membrane in the PAOI-V+VRT-325 condition, was also associated with impaired functional rescue. Indeed, the small rescue in CFTR_Inh172_-sensitive currents by VRT-325 treatment (LB+VRT-325) was abolished by PAO1-V exoproducts (Figures 4B,C).

Importantly, the Δ*lasR* mutation abrogated the inhibitory effect of PAO1-V, leading to similar levels of F508del-CFTR maturation (Figure 4A) and functional rescue (Figures 4B,C) by VRT-325 in LB+VRT-325 and PAO1-VΔ*lasR* +VRT-325 conditions. Similarly, quorum sensing inhibition of PAO1 cultures with HDMF prevented its deleterious action on CFTR maturation (Figure 5A) and currents (Figures 5B,C) in VRT-325-treated CFBE-ΔF508 cells.

The effect of HDMF-treated *P. aeruginosa* was also assessed on CFBE-ΔF508 cells rescued with the clinically used CFTR corrector Vx-809. As shown in Figure 6A, HDMF treatment restored the Vx-809 mediated CFTR maturation to levels similar to control condition (LB+Vx-809, without *P. aeruginosa* exoproducts). Moreover, HDMF treatment of PAO1 cultures (PAO1 HDMF+Vx-809 condition) counteracted the negative impact of *P. aeruginosa* (PAO1+Vx-809) on CFTR_Inh172_-sensitive currents after activation with Vx-770 and cAMP (Figures 6B,C).

Finally, we validated the beneficial effect of *P. aeruginosa* quorum sensing inhibition with HMDF on the CFTR functional rescue in differentiated primary airway epithelial cultures from 4 different CF patients homozygous for F508del mutations. Our data, presented individually for each tested patient, showed that despite variable levels of functional rescue of CFTR currents with Vx-809 among patients (LB+Vx-809 vs. LB), the inhibitory effect of PAO1 exoproducts (PAO1+Vx-809) was abrogated by HDMF (PAO1+HDMF+Vx-809) (Figure 7).

## Discussion

Our study first showed that exoproducts from *P. aeruginosa* laboratory and clinical strains with functional LasR (wild-type *lasR* allele) impaired both expression and function of wt-CFTR, as well as the functional rescue of F508del-CFTR by VRT-325 and Vx-809 correctors. However, mutations in a CF-adapted isolate or loss of LasR function in an engineered *lasR* mutant, led to a loss of *P. aeruginosa*'s inhibitory effect on CFTR. These data indicate that *P. aeruginosa* elicits different outcomes, as a function of their genotypic and phenotypic characteristics, on CFTR function in the airways of infected non-CF patients as well as on the efficacy of CFTR correctors in CF patients. Importantly, our data also unveiled that interfering with quorum sensing systems, and secondarily with virulence production, using a quorum sensing inhibitor, not only abrogated the negative impact of *P. aeruginosa* on wt-CFTR but also allowed to preserve the efficacy of CFTR correctors, despite the presence of *P. aeruginosa* infection.

Our data first demonstrated that CFBE-wt cells exposed for 24h to exoproducts from a clinical isolate (Early) or laboratory strains (PAO1 and PAO1-V) exhibited lower total protein expression of wt-CFTR and altered membrane localization, associated with reduced apical cAMP-activated CFTR_Inh172_-sensitive Cl^−^ currents. These observations are in agreement with our previous data on CFBE and primary AEC challenged with exoproducts from the *P. aeruginosa* CF clinical isolate PACF508 (Trinh et al., 2015). A dramatic decrease in CFTR total (Bomberger et al., 2011; Rubino et al., 2014; Saint-Criq et al., 2017) and membrane (Swiatecka-Urban et al., 2006; MacEachran et al., 2007; Bomberger et al., 2011) expression as well as Cl^−^ currents (Swiatecka-Urban et al., 2006; Saint-Criq et al., 2017) have also been reported after exposure to live PAO1 (Rubino et al., 2014) and PA14 (Swiatecka-Urban et al., 2006; MacEachran et al., 2007; Bomberger et al., 2011) laboratory strains or bacteria-free PAO1/PA14 filtrates containing exoproducts (Swiatecka-Urban et al., 2006; MacEachran et al., 2007; Saint-Criq et al., 2017). However, the effect of *P. aeruginosa* on CFTR function may be time- and cell-type dependent. Indeed, a previous study showed increased CFTR function after acute (15 min) challenge with PA14 in 2WT2 epithelial cells expressing wt-CFTR (Haenisch et al., 2010). A CFTR-dependent increase in airway surface liquid secretion has also been observed in swine trachea submucosal glands after a 35 min exposure to *P. aeruginosa* (Luan et al., 2014). These observations could be interpreted as an early response to bacterial infection by the airway epithelium, in an attempt to favor the clearance of pathogens through improved CFTR-dependent Cl^−^ and fluid secretion; whereas a longer exposure to *P. aeruginosa* elicits a negative effect on CFTR.

The mechanisms responsible for the observed decrease in CFTR expression and function may be multiple. Our previous study (Trinh et al., 2015) showed that exposure to PACF508 exoproducts decreased CFTR protein synthesis and enhanced protein degradation. The Stanton's group also reported a reduced number of CFTR channels at the apical membrane due to increased CFTR degradation in lysosomes and altered endocytic recycling in infectious conditions (Swiatecka-Urban et al., 2006; MacEachran et al., 2007; Bomberger et al., 2011). Several lines of evidence indicated that this effect may be mediated by the CFTR inhibitory factor (Cif, PA2394) from the outer membrane vesicles of *P. aeruginosa*, through a reduction of USP10-mediated deubiquitination of CFTR (MacEachran et al., 2007; Bomberger et al., 2011). Another study indicated that PAO1 inhibits CFTR endocytic recycling through a post-transcriptional modification of NHERF, by a mechanism dependent on bacterial pili and flagellin (Rubino et al., 2014). Furthermore, various secreted bacterial products may impair CFTR expression and function. A recent study (Saint-Criq et al., 2017) showed that LasB elastase in PAO1 secretome degrades CFTR and decreases CFTR activity. Our data also point toward a role for exoproducts under the control of quorum sensing, especially LasR. Indeed, *P. aeruginosa* strains carrying wild-type *lasR* alleles (PAO1, PAO1-V, Early) severely impaired CFTR, whereas an engineered (PAO1-V ΔlasR) mutant did not alter CFTR expression and function. Notably, CFTR impairment was also abolished with a spontaneous CF-adapted (Late) *lasR* mutant. This Late isolate harbors 68 different mutations compare to the Early, including in lasR and rhlR quorum sensing systems, as well as virulence factors and virulence regulators. Accordingly, we previously showed that the Late isolate exhibited reduced elastase/protease activities (Ruffin et al., 2016), which may explain why this isolate elicited less harmful impact on CFTR. Due to the number of mutations in the Late isolate, it is however not possible to ascertain whether the loss of deleterious effect on CFTR is due to the *lasR* mutation. LasR complementation in the Late strain would have been useful to test this hypothesis. Notably, the Late mutant exhibit reduced production of protease and elastase, as well as pyocyanine (Smith, 2006; Ruffin et al., 2016). The latter, a phenazine toxin, has been shown to down-regulate the vacuolar ATPase-dependent expression and apical membrane localization of CFTR (Kong et al., 2006). Our findings also provided the first proof of concept that a quorum sensing inhibitor (HDMF) decreased elastase production, which is directly regulated by LasR and abolished *P. aeruginosa*'s negative effect on CFTR. We are aware however that HDMF does not elicit a specific effect on the LasR quorum sensing, since it has been shown to inhibit the production of virulence factors regulated by the las, rhl and pqs quorum sensing systems (Choi et al., 2014). Altogether, work from our group and others indicate that various bacterial factors and cellular mechanisms likely contribute to the regulation of CFTR expression and function by *P. aeruginosa*.

Our study showed that exposure to PAO1 and PAO1-V exoproducts reduced F508del-CFTR maturation as well as cAMP- and Vx-770 stimulated CFTR currents in CFBE-ΔF508 and primary CF AEC treated with either VRT-325 or Vx-809 correctors. These data are in agreement with previous reports (Stanton et al., 2015; Trinh et al., 2015), including from our group, revealing that certain *P. aeruginosa* strains and CF clinical isolates may have a deleterious impact on CFTR functional rescue. In clinical studies of CFTR modulators, including CF patients infected/colonized with *P. aeruginosa*, we speculate that the negative effect of *P. aeruginosa* on CFTR rescue contribute to the lesser clinical benefits of CFTR modulators on lung function (Wainwright et al., 2015), compared to their expected *in vitro* activity established in pathogen free conditions.

*P. aeruginosa* undergo genetic adaptation during CF infection, leading to mutations associated with phenotypes such as loss of LasR quorum sensing signaling function and mucoidy (alginate overproduction). Results from our group (Trinh et al., 2015) and others (Stanton et al., 2015) indicated that both mucoid and non-mucoid *P. aeruginosa* isolates altered CFTR expression and rescue. In this study, we now show for the first time that *lasR* loss-of-function mutations as well as mutations in genes coding for virulence factors and virulence factors regulators [as found in the Late isolate (Smith, 2006)] abolished the deleterious impact of *P. aeruginosa* on CFTR. This observation thus indicated that the effect of *P. aeruginosa* on CFTR varies as a function of the infecting bacteria and may evolve over the course of the disease. This may thus explain, at least in part, the marked variability in clinical efficacy of CFTR modulators among patients, even when they harbor the same *CFTR* mutation (Durmowicz et al., 2013; Wainwright et al., 2015; Ramsey et al., 2017). Moreover, the response of AEC to CFTR correctors/potentiators may be even more difficult to predict as genetically and phenotypically heterogeneous *P. aeruginosa* populations co-exist in different regions of the lungs within the same patients (Workentine et al., 2013). The impact of *P. aeruginosa* on CFTR thus adds to other factors that may modulate the response to treatments in CF patients, including drug pharmacokinetics, the presence of complex CFTR alleles and modifier genes (Strug et al., 2016), the level of residual CFTR function, the influence of environmental factors and the lung disease severity at the time of treatment initiation.

Finally, our results unveiled that the inhibitory effect of PAO1 on CFTR maturation and functional rescue in Vx-809 and Vx-770 treated CFBE-ΔF508 can be significantly reduced by a treatment of *P. aeruginosa* with HDMF. Individual analysis of CFTR currents in primary CF human airway epithelial cell cultures then indicated that HDMF treatment abolished the deleterious impact of *P. aeruginosa* for all the 4 tested patients. We are aware that the concentration of HDMF used in that study is high. However, we previously verified that bacterial growth was not affected by this dose of HDMF, indicating that the observed effect was not due to an antimicrobial activity of HDMF on *P. aeruginosa* (Ruffin et al., 2016). Moreover, we verified that HDMF did not elicit any effect *per se* on CFTR, but reduced *P. aeruginosa* virulence (Supplementary Figure S1). Quorum sensing inhibitors, such as HDMF, have indeed been shown to limit virulence factor production and biofilm formation, and have been proposed as adjuvant therapy to antibiotics (Hurley et al., 2012; Bhardwaj et al., 2013; Kalia, 2013; Choi et al., 2014). Importantly, our data demonstrated that quorum sensing inhibitors may also counteract the harmful action of *P. aeruginosa* infection on the rescue of F508del-CFTR in CF AEC. Although additional studies are required to further develop this approach, our work provide the first proof of evidence that interfering with quorum sensing systems, especially LasR, could improve the effectiveness of CFTR-directed treatments in CF patients.

## Author contributions

EM, MR, DA, DN, and EB designed the study; EM, MR, DA, HM, SL, and GM performed the experiments; EM, MR, DA, HM, SL, DN, and EB analyzed and/or interpreted the data. EM and EB wrote the manuscript; MR, DA, and DN revised the manuscript for important intellectual content. All authors approved the final version of the manuscript and agree to be accountable for the content of the work.

### Conflict of interest statement

The authors declare that the research was conducted in the absence of any commercial or financial relationships that could be construed as a potential conflict of interest.
