# Supplementary figures and images for: Quorum Sensing Down-Regulation Counteracts the Negative Impact of Pseudomonas aeruginosa on CFTR Channel Expression, Function and Rescue in Human Airway Epithelial Cells
Source: Front Cell Infect Microbiol. 2017 Nov 10;7:470. doi: 10.3389/fcimb.2017.00470 (PMC5686086; doi:10.3389/fcimb.2017.00470)

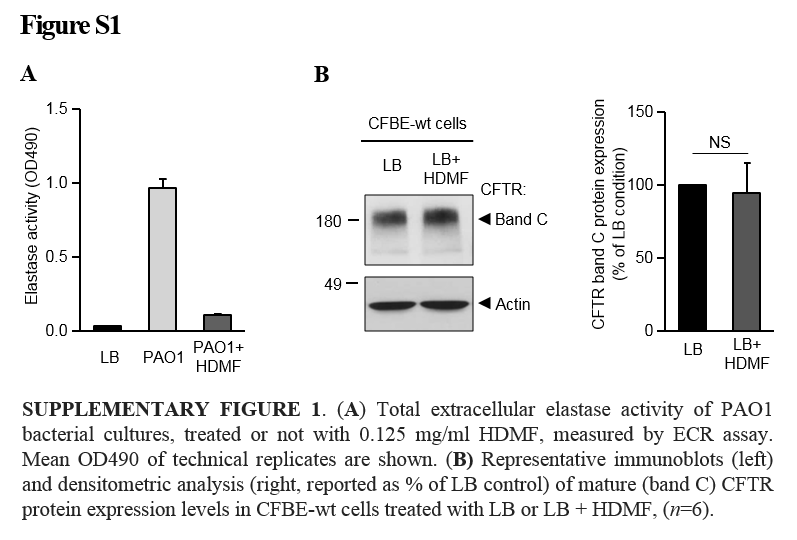

Supplement: Supplementary file 1 [file Image1.TIF]
